# Exploring Online Health Reviews to Monitor COVID-19 Public Health Responses in Alabama State Department of Corrections: Case Example

**DOI:** 10.2196/32591

**Published:** 2021-11-10

**Authors:** Pamela Valera, David Carmona, Sarah Malarkey, Noah Sinangil, Madelyn Owens, Asia Lefebre

**Affiliations:** 1 Department of Urban-Global Public Health Rutgers School of Public Health Newark, NJ United States

**Keywords:** Alabama, correctional facilities, COVID-19, online health reviews, review, monitoring, public health, policy, response, prison, United States, case study, formative, feasibility, acceptability, survey

## Abstract

**Background:**

COVID-19, caused by SARS-CoV-2, has devastated incarcerated people throughout the United States.

**Objective:**

The purpose of this study was to test the feasibility and acceptability of a COVID-19 Health Review for Correctional Facilities.

**Methods:**

The COVID-19 Health Review survey for the Department of Corrections was developed in Qualtrics to assess the following: (1) COVID-19 testing, (2) providing personal protective equipment, (3) vaccination procedures, (4) quarantine procedures, (5) COVID-19 mortality rates for inmates, (6) COVID-19 mortality rates for correctional officers and prison staff, (7) COVID-19 infection rates for inmates, (8) COVID-19 infection rates for correctional officers and prison staff, and (9) uptake of COVID-19 vaccines. The estimated time to review the Alabama State Department of Corrections COVID-19 responses on their website and complete the survey items was 45 minutes to 1 hour.

**Results:**

Of the 21 participants who completed the COVID-19 Health Review for Correctional Facilities survey, 48% (n=10) identified as female, 43% (n=9) identified as male, and 10% (n=2) identified as transgender. For race, 29% (n=6) self-identified as Black or African American, 24% (n=5) Asian, 24% (n=5) White, 5% (n=1) Pacific Islander or Native Hawaiian, and 19% (n=4) Other. In addition, 5 respondents self-identified as returning citizens. For COVID-19 review questions, the majority concluded that information on personal protective equipment was “poor” and “very poor,” information on COVID-19 testing was “fair” and above, information on COVID-19 death/infection rates between inmates and staff was “good” and “very good,” and information on vaccinations was “good” and “very good.” There was a significant difference observed (*P*=.03) between nonreturning citizens and returning citizens regarding the health grade review with respect to available information on COVID-19 infection rates.

**Conclusions:**

COVID-19 health reviews may provide an opportunity for the public to review the COVID-19 responses in correctional settings.

## Introduction

### Background

COVID-19, caused by SARS-CoV-2, has devastated people nationwide in the United States, particularly people who are incarcerated. There are 2.3 million incarcerated people in the United States that are imprisoned in various correctional institutions across the criminal justice system [1]. As of June 2021, nearly 400,000 infectious cases and close to 3000 deaths in prison were attributed to COVID-19 [2,3]. Furthermore, COVID-19 infection rates in prisons were found to be 5.5 times higher than that of the general US population [4], and mortality rates due to COVID-19 in correctional facilities were found to be 2.5 times higher than that of the general US population [4,5]. COVID-19 outbreaks across correctional settings, including Ohio’s Cook County Jail accounting for 15% of Chicago’s total COVID-19 cases [6] and Alabama’s COVID-19 outbreak in Ashville Jail with 37 inmates testing positive [7], exposed the deep structural and administrative problems with limited public health measures in place. The COVID-19 preventative measures of social distancing and quarantining are nearly impossible to implement as incarcerated people share common areas such as cell units, bathroom stalls, showers, and cafeterias, among others. These living conditions are often overcrowded and poorly ventilated [8-11].

Concurrently, correctional officers and other prison staff workers are susceptible to COVID-19 as they are in and out of facilities, which increases the likelihood of spreading and transmitting the virus [9,10,12,13]. The COVID-19 pandemic has further reduced the already declining number of correctional staff, severely impacting public health responses [13-15].

The incarcerated population is also aging and typically older; at least half are 50 years or older, report having a chronic condition, and are 2.5 times more likely to report a chronic illness than the general population [16]. COVID-19 typically has a greater impact on older people, particularly those with preexisting conditions [17]. The lack of inadequate and unequal access to health care services may further exacerbate the impact of COVID-19 on correctional settings and its incarcerated people [8,13].

### Understanding the State Department of Corrections’ Response to COVID-19

The State Department of Corrections’ response to COVID-19 was delayed during the early days of the pandemic due to the novelty of the disease [18]. When the Centers for Disease Control and Prevention (CDC) called for face masks and gloves to be worn when social distancing was impossible, the US Bureau of Prisons (BOP) confirmed that personal protective equipment (PPE) had been provided to all inmates and prison staff [19]. Yet, reports of unavailable PPE in various prisons and the national shortage of PPE resulted in the failed initial response to COVID-19 in prisons [19].

### BOP Rules and Regulations Concerning Inmate Transfers

The BOP stated that inmates could be transferred if they met three conditions: (1) passing a COVID-19 exit screening or not showing any symptoms, (2) being held for 14 days in BOP custody, and (3) notifying the BOP Emergency Operations Center before the transfer of an inmate [20]. Although prison visitations were prohibited, many incarcerated people continued to be transferred without proper COVID-19 testing [18]. Indeed, testing and contact tracing were relatively slow, causing infection cases to go undetected [18,21]. One of the more sought-after responses to combat COVID-19 was to reduce jail and prison populations by releasing incarcerated people, which would have slowed the spread and death of the prison population [22-24]. However, during the latter half of 2020, many prison systems such as those in Wyoming, Mississippi, Montana, and Hawaii were still at 90% or higher capacity [25].

### Online Health Reviews

Understanding how the US State Department of Corrections responded to the COVID-19 pandemic in prisons may help to build best practices regarding the detection and prevention of COVID-19. Hence, it is vital to evaluate and assess actions taken as part of a public health response to reduce the spread of COVID-19 in prisons. One public health approach that has been shown to connect people with vital information about local organizations is online health reviews.

Online health reviews are designed to help consumers find vetted local providers such as dentists, physicians, other specialty care providers, and many more. Online health reviews, or “health grades,” are generally used to review the quality of a physician’s or provider’s care or a patient’s visit. Over the past few years, there has been significant growth in the use and development of health reviews and ratings in the United States [26]. Online reviews such as Yelp or HealthGrades may provide the public an opportunity to monitor these venues and improve the state or local inspection efforts. Consumers who review their experience and leave reviews help others make informed decisions about the quality of care they may receive from the specific hospital or provider. In addition, studies have shown that online health reviews can help surgeons, physicians, and other medical providers improve their overall care and treatment of patients [26-28]. Most importantly, the increased usage of online reviews can also impact patient choice. Reviews that express a negative comment can influence another patient’s decision to seek medical care at that location [27].

### Online Reviews in Providing Patient Choices

Online health reviews may provide people with confidence that they are making informed choices by reading through the reviews about the business and helping to recommend their provider to friends and family [29]. When we look deeper into the content of reviews, patients often talk about relationships with their medical provider, suggesting that such interactions may be essential to a patient when leaving a review [30]. Health reviews undoubtedly play a vital role in sharing patient experience and even influencing patient choice. Although online health reviews may play a critical role in influencing a patient’s choice, reviews about correctional facilities’ public health response to COVID-19 are relatively unknown. Online health reviews could potentially improve COVID-19 responses in correctional facilities [31].

### Objective of the Present Study

Given the urgent need to prevent and reduce the spread of COVID-19 in correctional facilities, this project aims to further understand the COVID-19 response of one State Department of Corrections by developing a health review survey using the Alabama State Department of Corrections (ADOC) as a case study. The ADOC was selected because it has one of the highest COVID-19 mortality rates among incarcerated people in the United States [2,3] and has faced substantial challenges in providing adequate public health responses to the COVID-19 pandemic [32,33].

The ADOC has had 2085 positive cases and 68 fatalities from COVID-19 among inmates [34]. COVID-19 vaccinations have been administered to 48.06% (11,895/24,751) of incarcerated people housed in their facilities [34]. The ADOC’s website provides detailed information on preventative and protective measures, testing updates, and operation plans. However, the ADOC has failed to provide proper COVID-19 testing and PPE, reduce inmate populations, release medically vulnerable individuals and those near the end of their sentences, and publicly make data available on COVID-19 [31]. Moreover, the ADOC has been fraught with controversy in recent months for denying COVID-19 vaccines to its incarcerated people. Litigation is underway by the US Justice Department against the ADOC for unsanitary and disturbing trends in reports of violence that exacerbate their inadequate response to COVID-19 [32,33].

## Methods

### Survey

The COVID-19 Health Review survey for the Department of Corrections was developed in Qualtrics to assess the following: (1) COVID-19 testing, (2) providing PPE, (3) vaccination procedures, (4) quarantine procedures, (5) COVID-19 mortality rates for inmates, (6) COVID-19 mortality rates for correctional officers and prison staff, (7) COVID-19 infection rates for inmates, (8) COVID-19 infection rates for correctional officers and prison staff, and (9) uptake of COVID-19 vaccines. To better understand the information on the Department of Corrections websites, the survey included eight items that focused on the CDC’s public health measures. Textbox 1 provides the list of questions. Participants were then asked to respond to each item on a 6-point scale (“very poor,” “poor,” “fair,” “good,” “very good,” or “unknown”). The survey was anonymous and no personal information was collected. The estimated completion time of the survey ranged from 45 minutes to 1 hour.

**Questions related to public safety items for COVID-19 measures on the website.**

**ALABAMA: Please review the Department of Corrections (DOC) website and rank the information for each component. To access the DOC website, right-click “Alabama” and select “open link in a new tab.”**

1. Does the website showcase information about providing protective equipment to incarcerated persons?
2. Is there policy in place for the prison to engage with public health recommendations like social distancing?
3. Are there data that inform the public about COVID-19 death rates among incarcerated persons?
4. Are there data that inform the public about COVID-19 death rates for correctional officers and prison staff?
5. Are there data that describe COVID-19 infection rates for incarcerated persons?
6. Are there data that describe COVID-19 infection rates for correctional officers and prison staff?
7. Has anyone been vaccinated within the prison setting?

Note: Each of these items was set on a 6-point scale: “very poor,” “poor,” “fair,” “good,” “very good,” or “unknown”

### Recruitment of Survey Participants

Participants were recruited via social media using email from a Listserv, Twitter, and Instagram from April 2021 to July 2021. A total of 35 participants completed the survey.

### Statistical Analysis

Demographic characteristics and COVID-19 health review results are reported with frequencies and percentages. The results were then stratified by those who self-reported that they were returning citizens or people with a criminal justice history and those who were not returning citizens to identify any significant differences. Fisher exact tests [35] were performed for this analysis, as the data were not normally distributed.

## Results

Thirty-five participants completed the survey; after removing 14 observations due to incomplete data, 21 observations remained for final analyses. Of the 21 participants, five identified as returning citizens and 16 did not report criminal justice involvement. The basic characteristics of the participants and comparisons between returning and nonreturning citizens are summarized in Table 1.

**Table 1:** Demographic characteristics of the participants.

Variable		Total sample (N=21), n (%)^a^	Returning citizens (n=5), n (%)	Nonreturning citizens (n=16), n (%)^a^	*P* value^b^
**Age group (years)**					.07
	18-24	8 (38)	1 (20)	7 (44)	
	25-34	4 (19)	0 (0)	4 (25)	
	35-44	4 (19)	2 (40)	2 (13)	
	45-54	2 (10)	0 (0)	2 (13)	
	55-64	2 (10)	2 (40)	0 (0)	
	≥65	1 (5)	0 (0)	1 (6)	
**Gender**					.79
	Female	10 (48)	2 (40)	8 (50)	
	Male	9 (43)	3 (60)	6 (38)	
	Transgender	2 (10)	0 (0)	2 (13)	
**Race (check all that apply)**					.08
	White	5 (24)	0 (0)	5 (31)	
	Black	4 (19)	1 (20)	3 (19)	
	African American	2 (10)	1 (20)	1 (6)	
	Asian	5 (24)	0 (0)	5 (31)	
	Pacific Islander/Native Hawaiian	1 (5)	1 (20)	0 (0)	
	Other	4 (19)	2 (40)	2 (13)	
**Hispanic or Latin origin**					.55
	Yes	5 (24)	2 (40)	3 (19)	
	No	16 (76)	3 (60)	13 (81)	
**Highest level of education achieved**					.03
	High school	8 (38)	4 (80)	4 (25)	
	Bachelor’s degree	9 (43)	0 (0)	9 (56)	
	Master’s degree	2 (10)	0 (0)	2 (13)	
	PhD or higher	1 (5)	0 (0)	1 (6)	
	Trade school	1 (5)	1 (20)	0 (0)	
**Current employment status**					.12
	Employed full time	9 (43)	4 (80)	5 (31)	
	Employed part time	8 (38)	0 (0)	8 (50)	
	Seeking opportunities	4 (19)	1 (20)	3 (19)	

^a^Due to rounding, percentages may not add up to 100.

^b^Fisher exact test.

Of the 21 participants, about 40% (n=8) were 18-24 years old. The sample was racially diverse, with about 30% (n=6) being Black or African American, a quarter White, followed by Asian Americans, Pacific Islander or Native Hawaiian, and a sizable “other” race category. Nearly half of the participants reported their gender identity as female, followed by male, and two individuals identified as transgender. The majority (76%, n=16) of the sample did not identify as being of Hispanic or Latino origin. Nearly 80% (n=17) of the participants were either employed part time or full time. The highest level of education was the only significant association between returning citizens and nonreturning citizens (*P*=.03). Eighty percent (n=4) of the returning citizens’ highest degree of education achieved was high school compared to the majority (75%, n=12) of the nonreturning citizens earning a bachelor’s degree or higher. Table 2 compares the results of the COVID-19 Health Review items for the ADOC between returning and nonreturning citizens.

**Table 2:** Comparative COVID-19 grade results among returning citizens and nonreturning citizens.

Health Review item			Total sample (N=21), n (%)^a^	Returning citizens (n=5), n (%)		Nonreturning citizens (n=16), n (%)^a^		*P* value^b^
**Does the website provide information relating to COVID-19 testing?**								.94
	Very poor	2 (10)		1 (20)	1 (6)			
	Poor	3 (14)		1 (20)	2 (13)			
	Fair	5 (24)		1 (20)	4 (25)			
	Good	6 (29)		1 (20)	5 (31)			
	Very good	5 (24)		1 (20)	4 (25)			
	Unknown	0 (0)		0 (0)	0 (0)			
**Does the website showcase information about providing protective equipment to incarcerated persons?**								.16
	Very poor	4 (19)		2 (40)	2 (13)			
	Poor	7 (33)		1 (20)	6 (38)			
	Fair	1 (5)		1 (20)	0 (0)			
	Good	3 (14)		0 (0)	3 (19)			
	Very good	2 (10)		1 (20)	1 (6)			
	Unknown	4 (19)		0 (0)	4 (25)			
**Is there policy in place to facilitate vaccination for all incarcerated persons and prison staff?**								.69
	Very poor	3 (14)		1 (20)	2 (13)			
	Poor	3 (14)		1 (20)	2 (13)			
	Fair	6 (29)		2 (40)	4 (25)			
	Good	4 (19)		0 (0)	4 (25)			
	Very good	2 (10)		1 (20)	1 (6)			
	Unknown	3 (14)		0 (0)	3 (19)			
**Is there policy in place for the prison to engage with public health recommendations like social distancing?**								.29
	Very poor	3 (14)		1 (20)	2 (13)			
	Poor	7 (33)		1 (20)	6 (38)			
	Fair	3 (14)		2 (40)	1 (6)			
	Good	2 (10)		1 (20)	1 (6)			
	Very good	2 (10)		0 (0)	2 (13)			
	Unknown	4 (19)		0 (0)	4 (25)			
**Are there data that inform the public about COVID-19 death rates for incarcerated persons?**								.51
	Very poor	1 (5)		0 (0)	1 (6)			
	Poor	3 (14)		2 (40)	1 (6)			
	Fair	2 (10)		0 (0)	2 (13)			
	Good	6 (29)		1 (20)	5 (31)			
	Very good	9 (43)		2 (40)	7 (44)			
	Unknown	0 (0)		0 (0)	0 (0)			
**Are there data that inform the public about COVID-19 death rates for correctional officers and prison staff?**								.09
	Very poor	1 (5)		0 (0)	1 (6)			
	Poor	3 (14)		2 (40)	1 (6)			
	Fair	3 (14)		2 (40)	1 (6)			
	Good	8 (38)		1 (20)	7 (44)			
	Very good	5 (24)		0 (0)	5 (31)			
	Unknown	1 (5)		0 (0)	1 (6)			
**Are there data that describe COVID-19 infection rates for incarcerated persons?**								.03
	Very poor	1 (5)		1 (20)	0 (0)			
	Poor	4 (19)		2 (40)	2 (13)			
	Fair	1 (5)		1 (20)	0 (0)			
	Good	8 (38)		0 (0)	8 (50)			
	Very good	6 (29)		1 (20)	5 (31)			
	Unknown	1 (5)		0 (0)	1 (6)			
**Are there data that describe COVID-19 infection rates for correctional officers and prison staff?**								.07
	Very poor	1 (5)		1 (20)	0 (0)			
	Poor	4 (19)		2 (40)	2 (13)			
	Fair	1 (5)		1 (20)	0 (0)			
	Good	9 (43)		1 (20)	8 (50)			
	Very good	4 (19)		0 (0)	4 (25)			
	Unknown	2 (10)		0 (0)	2 (13)			
**Uptake of COVID-19 vaccines: has anyone been vaccinated within the prison setting?**								.30
	Very poor	0 (0)		0 (0)	0 (0)			
	Poor	3 (14)		2 (40)	1 (6)			
	Fair	2 (10)		0 (0)	2 (13)			
	Good	7 (33)		1 (20)	6 (38)			
	Very good	7 (33)		1 (20)	6 (38)			
	Unknown	2 (10)		1 (20)	1 (6)			

^a^Due to rounding, percentages may not add up to 100.

^b^Fisher exact test.

Sixteen respondents reported that the information provided on the website related to COVID-19 testing was “fair” and above. Despite having “fair” information about COVID-19 testing, half of the respondents (n=11) reported the details on providing PPE to incarcerated persons as “poor” and “very poor.” Furthermore, approximately half (n=12) of the respondents reported the information about policies to facilitate vaccinations for all incarcerated persons and prison staff as “fair” and above. Approximately 10 of the respondents reported guidelines for the prison to engage with public health recommendations as “very poor” and “poor.” Approximately 15 participants of the sample reported information on COVID-19 death rates among incarcerated persons as “good” and “very good.” A total of 13 participants provided the same rating with respect to data about correctional officers and prison staff.

With regard to COVID-19 infection rates, most respondents reported that the ADOC’s COVID-19 website provided “good” and “very good” data for both incarcerated persons and correctional officers. Two-thirds (n=14) of the sample reported that the information provided about whether people were vaccinated within the prison setting was “good” and “very good.” The only significant difference between returning and nonreturning citizens was whether the data included on the ADOC website describe incarcerated people’s COVID-19 infection rates (*P*=0.03): 80% (n=4) of the returning citizens reported the data included as “fair” and below compared to 75% (n=12) of the nonreturning citizens reporting the data included as “good” and “very good.”

Participants were also asked to provide feedback about the ADOC COVID-19 website. Example quotes of some of this feedback are provided in Textbox 2.

**Representative feedback about the Alabama Department of Corrections' COVID-19 website.**

“They had the basic info. The more in-depth information was difficult to find.”

“No information on COVID-19 procedures.”

“I quickly found all other information. This took me about 3 minutes. The COVID information was very clear, but in English only.”

“The website did not provide information on PPE [personal protective equipment] equipment and how vaccines will be given to inmates. COVID-19 preparedness is shown in a single document that describes everything but specific quarantine procedures. The website had a dashboard, so it was fairly quick to find basic information on COVID-19 such as rates.”

“The information is there, but little. And, it seems that they are going to start the vaccine. I looked a lot because I was looking for information about PPE.”

## Discussion

### Principal Findings

This online COVID-19 public health review in a correctional setting provides an opportunity to examine the current operational practices at the population level in response to the COVID-19 pandemic. This is the first study to explore the role of digital health response to outbreaks, vaccines, and pandemics that focuses on correctional facilities, which may help to reduce the deadly impact of COVID-19 on the lives of marginalized communities. Our findings build on emerging literature that describes online health review topics that could potentially be applied in public health responses to COVID-19 [29,36,37].

We believe that these results may serve as an essential reference point for policymakers and advocates to understand the impact and relevance of COVID-19 online health reviews, and to prioritize resources and efforts to address the challenges presented by COVID-19 in correctional settings. For example, most participants felt that the reported PPE information provided on the ADOC website was “poor” and “very poor.” This implies that information provided to the public about PPE may be limited. At the same time, there was a significant difference (*P*=.03) observed between nonreturning citizens and returning citizens on the quality of information about COVID-19 infection cases displayed on the ADOC website. The majority of returning citizens found this to be “fair.” In contrast, the participants without a criminal justice history found the information to be “good” and “very good.” Although participants noted that COVID-19 public health measures were available on the ADOC website, this information was only available in English. An official website of the US government should be available in multiple languages.

Furthermore, using health grades or online reviews to understand the mitigation and adaptation responses to COVID-19 is critical to prevent the infection and mortality rates in correctional settings. This would allow for additional oversight and place pressure on the State Department of Corrections to do the right thing, especially during a pandemic where correctional officers and incarcerated persons are directly impacted. As previously mentioned, there has been no use of health grades or online reviews in prison systems to date.

Our study presents an opportunity to enter a new landscape of grading or reviewing correctional facility responses toward COVID-19. The existing scientific literature on health grades and online reviews has stressed how reviews can influence people’s perceptions and choices regarding seeking care and even recommending care [27,29] while also having the potential to improve the quality of care and treatment [26-28]. Our work builds upon the current literature by extending the potential of influencing people’s choices and perceptions toward how correctional facilities are handling COVID-19 and improving these same correctional facilities to better respond to the impact of COVID-19.

### Implications for Future Research

Given how critical online reviews can be, there is much potential in using online reviews to cover more aspects of correctional facilities. For example, although we specifically focused on the ADOC in this study, there may be potential to create a nationwide health grading system covering every Department of Corrections. For example, the web-based company HealthGrades has a database on all doctors in the United States and includes patient reviews for every doctor [38]. In a similar vein, we may create an online database that keeps track of all COVID-19 responses across all Departments of Corrections in the United States. This information is vital for frontline workers at correctional facilities to improve and provide a safer and secure service for incarcerated people.

### Limitations

Our survey results have several limitations that must be acknowledged. First, not all recruited participants were able to complete the COVID-19 correctional health survey, resulting in incomplete and missing data. The length of the survey is also a limitation, as the 1-hour completion time may have impacted our participants’ ability to examine the ADOC website properly.

### Conclusions

The COVID-19 pandemic is still ongoing and new technologies are needed to evaluate the impact of COVID-19 in correctional settings. There is added value to examine how online health reviews can be used to understand the COVID-19 pandemic response in correctional facilities. Online health reviews offer tools to inform the public about how the Department of Corrections supports a public health pandemic response that saves lives.

## Abbreviations

ADOC: Alabama State Department of Corrections
BOP: US Bureau of Prisons
CDC: Centers for Disease Control and Prevention
PPE: personal protective equipment
